# *FosSahul* 2.0, an updated database for the Late Quaternary fossil records of Sahul

**DOI:** 10.1038/s41597-019-0267-3

**Published:** 2019-11-19

**Authors:** Katharina J. Peters, Frédérik Saltré, Tobias Friedrich, Zenobia Jacobs, Rachel Wood, Matthew McDowell, Sean Ulm, Corey J. A. Bradshaw

**Affiliations:** 1 Global Ecology Lab, College of Science and Engineering and ARC Centre of Excellence for Australian Biodiversity and Heritage, Flinders University, GPO Box 2100, Adelaide, South Australia 5001 Australia; 2 Department of Oceanography, University of Hawai’i at Mānoa, Honolulu, Hawaii USA; 3 Centre for Archaeological Science, School of Earth, Atmospheric and Life Sciences and ARC Centre of Excellence for Australian Biodiversity and Heritage, University of Wollongong, Wollongong, New South Wales Australia; 4 Radiocarbon Facility, Research School of Earth Sciences, The Australian National University, Canberra, ACT 2601 Australia; 5 School of Archaeology and Anthropology, The Australian National University, Canberra, ACT 2601 Australia; 6 Dynamics of Eco-Evolutionary Patterns and ARC Centre of Excellence for Australian Biodiversity and Heritage, University of Tasmania, Tasmania, 7001 Australia; 7 ARC Centre of Excellence for Australian Biodiversity and Heritage, College of Arts, Society and Education, James Cook University, PO Box 6811, Cairns, Queensland 4870 Australia

**Keywords:** Palaeontology, Biodiversity, Palaeoecology

## Abstract

The 2016 version of the *FosSahul* database compiled non-human vertebrate megafauna fossil ages from Sahul published up to 2013 in a standardized format. Its purpose was to create a publicly available, centralized, and comprehensive database for palaeoecological investigations of the continent. Such databases require regular updates and improvements to reflect recent scientific findings. Here we present an updated *FosSahul* (2.0) containing 11,871 dated non-human vertebrate fossil records from the Late Quaternary published up to 2018. Furthermore, we have extended the information captured in the database to include methodological details and have developed an algorithm to automate the quality-rating process. The algorithm makes the quality-rating more transparent and easier to reproduce, facilitating future database extensions and dissemination. *FosSahul* has already enabled several palaeoecological analyses, and its updated version will continue to provide a centralized organisation of Sahul’s fossil records. As an example of an application of the database, we present the temporal pattern in megafauna genus richness inferred from available data in relation to palaeoclimate indices over the past 180,000 years.

## Introduction

The study of fossils provides essential insights into past ecological processes^1^, and the origins and evolution of species through time^2,3^. With the advancement of dating methods, the number of reliably dated fossil records — i.e., those with quantified confidence intervals encompassing the true age of the specimen^4^ — is steadily increasing. However, to apply the information of these records in comprehensive analyses, they first need to be compiled and organized in a standardized and detailed framework. The development of the *FosSahul* database^5,6^ unified and quality-controlled fossil specimens across the Sahul region (i.e., mainland Australia, Tasmania, New Guinea, joined at times of lower sea levels), and has opened many opportunities for new, broad-scale analyses of ecological processes^7^. For example, since its compilation, the database has provided a robust source of data to investigate extinction drivers at both continental and regional scales in Australia^8–10^, and to identify new areas for potential fossil discovery^11^.

To maintain *FosSahul*’s utility, given the growing wealth of new fossil data being published each year, regular updates and maintenance are essential to ensure this database incorporates the latest fossil discoveries and remains relevant. Furthermore, *FosSahul* includes its own quality-rating scheme based on a two-stage set of objective criteria^4^. Because dating methods will continue to improve and new research will provide more reliable fossil ages of both existing and newly discovered fossils, it is important to have a fully transparent and easily reproducible quality-rating method.

In this paper, we provide the latest updated version of the *FosSahul* database (*FosSahul* 2.0) and present an automated algorithm that runs the two-stage quality assessment^4^ on dated fossil records in the database. We describe this new feature and demonstrate the versatility and applicability of *FosSahul* by using the resulting high-quality ages to calculate a megafauna (>44 kg adult mass^12^) vertebrate richness indicator^13^ for the last 180,000 years across Australia, and visually link this to palaeoclimate metrics (i.e., mean annual temperature and precipitation anomalies and velocity, see *Methods*) based on climate hindcasts from the LOVECLIM Earth-system model^14^.

## Results

### Database update

**Fig. 1:**
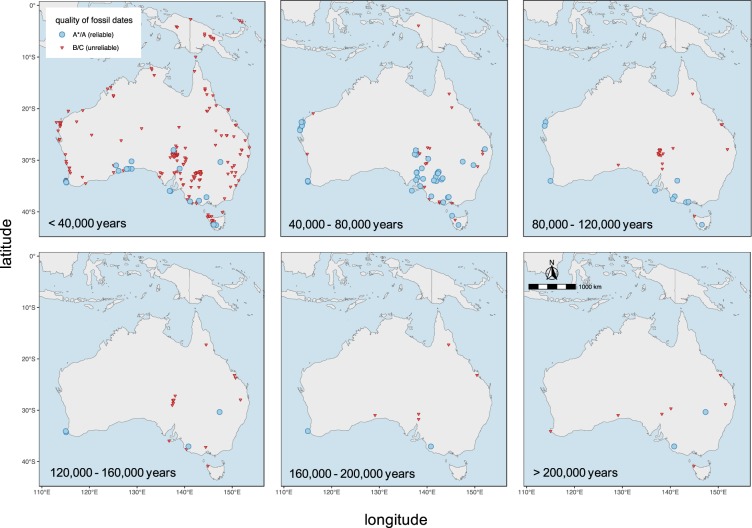
Distribution of dated non-human vertebrate fossil records in the Sahul region for different time periods of the Late Quaternary (up to 897,000 years ago) rated as reliable (A* or A; blue circles) and unreliable (B or C; red triangles).

Compared to its originally published version, the updated *FosSahul* (2.0) database^15^ contains ~ 28% more dated fossil records (11,871 in total, see Table 1) compiled from 160 published sources (previously 144). Of these records, 27% are fossils of megafauna. The fossil records stem from 605 deposits (an increase of 67%) and encompass 1% more species and genera than *FosSahul* 1.0. Of all records, 14% (1,626) received a quality rating of A* or A. These 1,626 records were located on the Australian mainland, Tasmania and Kangaroo Island, with a concentration in south-eastern Australia, in the coastal regions of Western Australia and along the Great Australian Bight (Fig. 1). There are no reliable records (B and below) for central and northern Australia and the rest of Sahul, and all reliable records older than 120,000 years are concentrated in three locations (Leeuwin–Naturaliste Region in Western Australia, Naracoorte in South Australia, and Cuddie Springs in New South Wales) (Fig. 1). Ages rated B or C can still be useful, for example, in analyses only requiring information on the presence or absence of species^11^, and so we retained these in the database.

**Table 1:** Summary metrics for the original (*FosSahul* 1.0) and the updated (*FosSahul* 2.0) versions of the database, including the % difference between the two. Reliability of ages is based on the quality-rating criteria established in Rodríguez-Rey *et al*.^4^.

	*FosSahul* 1.0	*FosSahul* 2.0	% difference
fossil records	9,302	11,871	+28
megafauna records	2,559	3,190	+25
genera	213 (215)*	217	+1
species	345 (478)*	354	+1
sources	144	160	+11
deposits	363	605	+67
reliable ages (Α* or Α)	2,422	1,626	−33

*Numbers in brackets were published originally, but they contained errors and so we corrected them here.

### Vertebrate richness and number of records in relation to climate

The corrected sampled-in-bin diversity index^13^ applied to high-quality megafauna ages of *FosSahul* 2.0 shows an increase (from 1.5 to 10; Fig. 3, top panel) in diversity of megafauna specimens until ~ 90,000 years ago, followed by a decrease until ~ 30,000 years ago when mean annual temperature slightly increased (+1 °C) after a drop of ~4 °C between 80,000 and 60,000 years ago. This variation in temperature seems to be part of a climate cycle that occurred also between 200,000 and 80,000 years ago. Such a climatic cycle is present in the mean annual precipitation outputs showing a succession of increase/decrease in precipitation approximatively every 20,000 years. Both temperature and precipitation velocity had low variation (i.e., <40 and 8 m year^−1^, respectively) across Sahul for the last 200,000 years. Although only ranging from 100,000 to 30,000 years ago, the diversity index of the southeast Australian subsample follows the same trend as that from the complete dataset, indicating that the richness estimates are probably still indicative of real trends despite the spatial bias potentially caused by the uneven distribution of fossil records in the full dataset.

## Discussion

**Fig. 2:**
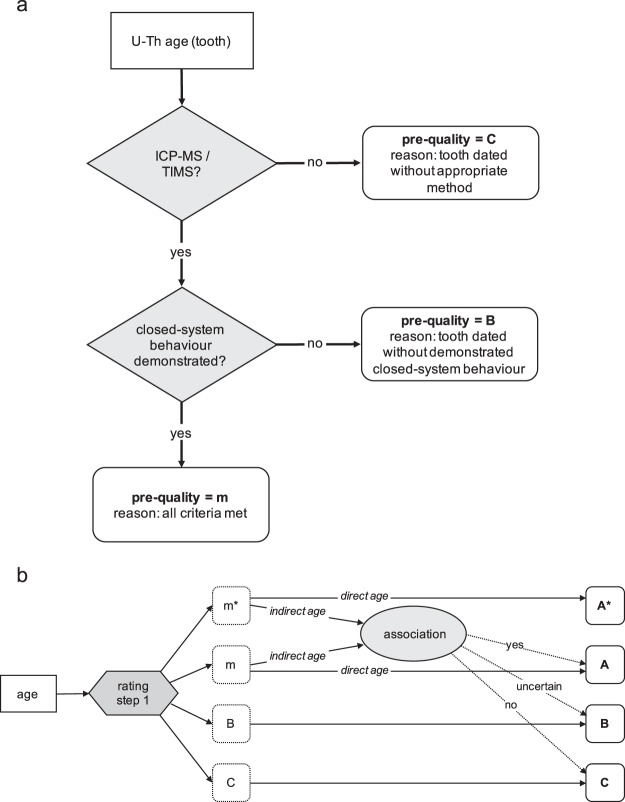
Flowchart presenting the quality-rating method implemented by the automated algorithm. Panel (a) shows the first step of the rating procedure using the example of an uranium-thorium series (U-Th) age for teeth, panel (b) shows the second step. Based on the pre-quality rating received in the first step (**a**), ages are given a final rating depending on whether they are direct or indirect ages and (only for indirect ages) their association with the dated material. Panel (b) has been adapted from Rodríguez-Rey *et al*.^4^.

The updated and extended *FosSahul* database (*FosSahul* 2.0) now contains a total of 11,871 dated non-human vertebrate fossil records from the Quaternary, originating from 160 sources published up to 2018. Compared to *FosSahul* 1.0, the updated version contains fewer records with a high-quality rating. The main reason for this is that the previous version of the database was rated ‘manually’ without the standardized consistency of the now-implemented algorithm. Therefore, although the authors relied on expert consultation for the quality ratings provided in *FosSahul* 1.0, not all of the information used for each record was consistently recorded in the first version of the database. This was a weakness of *FosSahul* 1.0, and the main reason for our extension of the database to include additional, detailed information, and the development of the R code to do the rating objectively. The new database is now accompanied by an algorithm (Fig. 2) that automates the previously developed quality-rating procedure for fossil ages^4^.

**Fig. 3:**
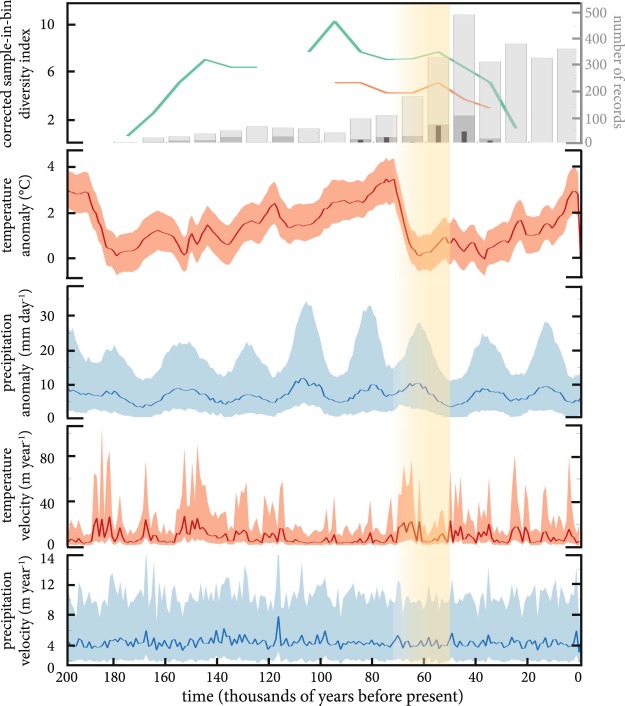
Genus-level corrected, sampled-in-bin diversity index calculated from *FosSahul* 2.0 high-quality ages (i.e., scored A* and A) for megafauna specimens (from Sahul = green; from south-eastern Australia = orange), number of megafauna records (based on the full dataset = light grey; high-quality ages only = dark grey; high-quality ages for south-eastern Australia only = thin black bars), mean annual temperature (°C) and precipitation anomaly (mm day^−1^) relative to the present day, temperature velocity (m year^−1^), and precipitation velocity (m year^−1^) across time (in thousands of years before present). Both the ‘corrected, sampled-in-bin diversity index’ and the ‘number of records’ are calculated using 10,000-year time increments, with the oldest records dated to 180,000 years before present. Climate variable plots show the median value (solid line), and the 25^th^ and 75^th^ percentiles (light shading) calculated across Sahul. Yellow shading represents putative arrival window (including uncertainties) of humans in Sahul, see Bradshaw *et al*.^41^ for discussion.

While the algorithm is tailored to *FosSahul*, it could easily be adapted to automate similar ratings on other databases. Although this algorithm has been designed to be user-friendly and to make the quality-rating process easier and less subjective, the user still has to make decisions in the process that require a sound understanding of the specific dating techniques and the quality-rating procedure itself. We therefore recommend that experts should be involved to avoid drawing misinformed conclusions regarding the quality of any age estimate. For future versions of *FosSahul*, the algorithm will be highly useful to non-specialists to rate the reliability of ages for new records that will be added to the database as new sources are published. Furthermore, it will allow a rapid recalculation of ratings as geochronologists reassess which methods and quality-assurance indicators are appropriate. This will facilitate the process of maintaining *FosSahul* as a database that reflects current scientific findings. The fact that fossil records are not evenly distributed across the continent underlines the need for new fossil discoveries, as well as improved dating methods and re-dating of existing fossils.

Climate change and/or the arrival of humans have been linked to an increase in megafauna extinctions in Sahul^8,10,16–21^, but there are many more elements that determine vertebrate richness across such broad spatial scales^22–24^. Therefore, it is essential to consider multiple potential drivers simultaneously, as well as the spatial patterns of biodiversity turnover when making conclusions about palaeoecological trends through time^10^.

In terms of climate, previous studies have highlighted that palaeoclimate trends in Australia are dominated by temporal variation in precipitation and/or aridity rather than temperature^25^, with important implications for extinctions — indeed, a decline in water levels at Lake Eyre and Lake Frome between 50,000 and 40,000 years before present were possibly related to local megafauna extinctions^26^, and Dortch *et al*.^27^ invoked drought as a cause of extinctions for *Macropus* spp. in south-eastern Australia. Spatial or temporal variation in precipitation has also been used to predict patterns in plant e.g.^28,29^ and mammal e.g.^30,31^ species richness, also across a wide range of latitudes^32^. Furthermore, water variables are strongly associated with vertebrate richness in regions with predominantly warm climates^32^. Climate stability has previously been associated with high biodiversity, given that environmental stability tends to minimize extinction and facilitate speciation through specialization and divergent selection^33,34^. However, given that the data in *FosSahul* only cover the last ~ 180,000 years at a continental scale, it is understandable that gross megafauna diversity patterns might not be explained by variation in climate stability over time^8,35^. Furthermore, here we only show patterns in vertebrate records at the broadest of scales, and climate conditions at finer scales potentially explain additional drivers of extinction patterns^10^ (Fig. 1). Although the current version of *FosSahul* does not contain records for investigating a finer-scale variation in diversity (at least not before 80,000 years ago), this is nevertheless an example of the type of analyses for which databases such as *FosSahul* can be used, particularly as more high-quality ages become available.

Overall, our study showcases the utility of the *FosSahul* database and highlights the importance of comprehensive databases for palaeoecological investigation. We emphasize that *FosSahul* is open to improvement and updates, and we encourage users to provide feedback on the database and the quality-rating algorithm.

## Methods

### Fossil data records

The original *FosSahul* database^5^ compiled Pleistocene to Holocene ages for non-human vertebrate fossil records from the Sahul region published in the scientific literature until 2013. When the database was assembled, Rodríguez-Rey *et al*.^4^ established a set of criteria to provide objective quality-rating criteria for fossil ages. We searched the literature for new fossil ages published from 2013 onwards and have updated the existing *FosSahul* website with these new records. Following the methods described in Rodríguez-Rey *et al*.^5^, we focused our literature search on megafauna records only because the collation of these was the original purpose of *FosSahul*. We only included non-megafauna vertebrate fossils when published alongside megafauna fossil dates. While these non-megafauna records are still useful, this part of the database was compiled opportunistically and is therefore not a complete representation of the available data. It is possible that a future version of *FosSahul* will include all fauna data; however, we have no such imminent plans.

### Automated quality-rating algorithm

We developed an algorithm to automate the two-step quality rating (Fig. 2b) described in Rodríguez-Rey *et al*.^4^ and Rodríguez-Rey *et al*.^5^. While Rodríguez-Rey *et al*.^5^ established the quality-rating system and compiled the initial *FosSahul* database, quality-rating had to be done manually^5^. To reduce errors and to make the dating process more objective, reproducible and transparent, we extended the database to include the necessary fields providing more detail on dating techniques (Online-only Table 3) to run the rating automatically based on the quality criteria described in Rodríguez-Rey *et al*.^4^ (Table 2, Fig. 2).

**Table 2:** Data quality rating generated by the algorithm.

Field	Description
pre-quality	m*, m, B, C
quality	A* (highly reliable), A (reliable), B (unreliable), C (highly unreliable)
reason	reason for pre-quality rating (e.g., ‘inadequate pre-treatment’)
quality reason	for A* and A ages, reason for quality rating; for all other ages, blank

In the first step, the algorithm rates the quality of the dating procedure based on the dated material (e.g., bone, charcoal, quartz grains) and the technique (e.g., ^14^C radiocarbon dating, amino acid racemization, luminescence dating, electron spin resonance, uranium-thorium series), including pre-treatments (if applicable). During this step, ages are assigned a pre-quality category (m*, m, B or C). For ages rated B or C, this rating is final. For ages that receive a pre-quality rating of m* or m, the second step of the algorithm rates these ages based on the association of the dated material with the remains of the target species^4,5^. For direct ages where the dated material is from the target species, the association is clear and the final rating is A* or A. For indirect ages, the association is assessed based on their specific depositional context (certain association = A; uncertain association = B; no or unknown association = C), with A being the highest possible rating for indirect ages (Table 2).

### Climate variables

We used a climate reconstruction based on the three-dimensional Earth-system model LOVECLIM^14,36^. The model includes representations of the atmosphere, ocean, sea ice, ice sheets, the carbon cycle, land surface, as well as a vegetation model that simulates the dynamics of two terrestrial plant functional types — trees and grasses. We simulated 1000-year average climates over the past 180,000 years using LOVECLIM and downscaled the output resolution to 1×1° using bilinear interpolation. For each temporal snapshot and grid cell, we extracted annual mean precipitation, and mean annual temperature. We then calculated the anomaly of each variable relative to present day.

### Index of vertebrate diversity in the database and climate-change metrics

Using only the high-quality fossil ages (A* and A), we calibrated the radiocarbon ages using the SHCal13 curve^37^ in OxCal 4.3^38^ and calculated a corrected sampled-in-bin diversity index (to genus level at the Australian continental scale) using the R package divDyn^13^ as a temporal measure of taxonomic richness represented in the database. This metric uses the richness per bin, but includes a measure of sampling completeness based on consecutive time bins as a correction for missing species that should have been present^39^. However, given the constraints of fossil preservation and patchy distribution of palaeontological excavation sites, the calculated richness index is likely still biased by uneven sampling effort. We used 10,000-year time increments with the oldest records dated to 180,000 years before present. Although the database contains fossil records for other areas of the Sahul region, none of those received a high-quality rating. Our analysis therefore only focuses on the Australian continent. To limit the spatial bias caused by the uneven distribution of fossil records here, we also analysed a subset of the data from south-eastern Australia (longitude < 130 E, latitude > 24 S) that has a more even distribution of records.

Climate anomaly (of a given climate variable relative to the present day) is one of the most common metrics used to describe exposure to climate change, but climate velocity is likely more biologically relevant than climate anomalies because velocity accounts for regional changes in climate and the ability of topographic heterogeneity to buffer biota against these changes^40^. Climate velocity describes the rate and direction that an organism would need to migrate to maintain an isocline of temperature and precipitation variables^40^. This proxy is often considered synonymous with the rate of climate displacement for a species, such that the higher the velocity, the less probable a species will keep pace with its shifting climatic niche and thus survive. We calculated vectors of climate velocity (m year^−1^) for mean annual temperature (°C) and mean annual precipitation (mm day^−1^) following established methods^40^ from the palaeoclimate reconstruction provided by LOVECLIM over the last 180,000 years. We divided the rate of climate change through time (°C year^−1^) by the spatial gradient in climate at that location (°C m^−1^).

## Data Availability

Data are available on figshare^15^ (10.6084/m9.figshare.8796944) and on the Global Ecology Flinders GitHub repository (https://github.com/GlobalEcologyFlinders/FosSahul).

## Online-only Table

**Online-only Table 1:** New fields added to *FosSahul* to provide necessary detail for the algorithm to automate the quality rating. ^14^C = radiocarbon; AAR = amino acid racemisation; U-Th = uranium-thorium; ESR = electron spin resonance; LD = luminescence dating.

Field	Sub field	Description
^14^C	pretreatment details	original information about pre-treatment from paper (detailed)
	pretreatment	short description of pre-treatment procedures (e.g., ‘acid wash”, ‘ABA’, etc)
	extraction problems	details on problems with extractions
	contamination	details on any contamination issues (‘yes’, ‘possible’, ‘likely’)
	C:N ratio	for radiocarbon ages of bone collagen and dentine collagen, give C:N ratio for specific sample
	N%	for radiocarbon ages of bone collagen and dentine collagen, give N% for specific sample
	x-ray diffraction	for radiocarbon ages of corals and shells, if x-ray diffraction done and showed no recrystallisation (‘yes’ or ‘no’)
AAR	thermal history	if thermal history is ‘burnt’, ‘unknown’ or ‘fine’
	closed-system behaviour	whether material demonstrated closed-system behaviour (‘yes’ or ‘no’)
	replicated with low uncertainty	whether multiple analyses were replicated with low uncertainties (‘yes’ or ‘no’)
	reliable calibration	whether reliable calibration was done using independent dating techniques (‘yes’ or ‘no’)
U-Th	pretreatment	short description of pre-treatment procedures (e.g., ‘grinding and acid etching’, etc)
	closed-system behaviour	for ages for teeth (dentine) and bone, if closed-system behaviour demonstrated by U-series profiling and modelling based on continuous profiles, or spot sampling using laser ablation (‘yes’ or ‘no’)
	detrital correction	uranium-series ages for closed system of no body remains (e.g., speleothems, corals, calcite within bones etc), whether correction made for detrital thorium contamination (‘yes’ or ‘no’)
ESR	internal dose rate <10%?	whether the internal dose rate is <10%, ‘yes’ or ‘no’
	gamma dose rate	whether the gamma dose rate was measured ‘in situ’ or ‘assumed’
LD	equivalent dose measurement	whether the equivalent dose is based on ‘single-grain’, ‘single-aliquot’ or ‘multiple-aliquot’ measurements
	bleaching status (equivalent dose distribution pattern)	Whether equivalent dose distributions display evidence that mineral grains were ‘adequately’ or ‘partially’ exposed to sunlight prior to deposition or ‘no’ information provided
	equivalent dose model	For single grain equivalent dose measurements, a specific model was used to describe the distribution and calculate a final value (‘yes’) or no model was used because the distribution suggest significant mixing (‘no’)

## Code availability

Code is available on figshare^15^ (10.6084/m9.figshare.8796944) and on the Global Ecology Flinders GitHub repository (https://github.com/GlobalEcologyFlinders/FosSahul).
