# Addendum: *FosSahul* 2.0, an updated database for the Late Quaternary fossil records of Sahul

**DOI:** 10.1038/s41597-021-00918-7

**Published:** 2021-05-13

**Authors:** Katharina J. Peters, Frédérik Saltré, Tobias Friedrich, Zenobia Jacobs, Rachel Wood, Matthew McDowell, Sean Ulm, Corey J. A. Bradshaw

**Affiliations:** 1 Global Ecology Lab, College of Science and Engineering and ARC Centre of Excellence for Australian Biodiversity and Heritage, Flinders University, GPO Box 2100, Adelaide, South Australia 5001 Australia; 2 Department of Oceanography, University of Hawai’i at Mānoa, Honolulu, Hawaii USA; 3 Centre for Archaeological Science, School of Earth, Atmospheric and Life Sciences and ARC Centre of Excellence for Australian Biodiversity and Heritage, University of Wollongong, Wollongong, New South Wales Australia; 4 Radiocarbon Facility, Research School of Earth Sciences, The Australian National University, Canberra, ACT 2601 Australia; 5 School of Archaeology and Anthropology, The Australian National University, Canberra, ACT 2601 Australia; 6 Dynamics of Eco-Evolutionary Patterns and ARC Centre of Excellence for Australian Biodiversity and Heritage, University of Tasmania, Tasmania, 7001 Australia; 7 ARC Centre of Excellence for Australian Biodiversity and Heritage, College of Arts, Society and Education, James Cook University, PO Box 6811, Cairns, Queensland 4870 Australia

Addendum to: *Scientific Data* 10.1038/s41597-019-0267-3, published online 19 November 2019

The original publication described *FosSahul* 2.0, the updated version of the *FosSahul* database comprising collated and quality-rated megafauna fossil ages of the Late Quaternary from Sahul, as well as R code to run the algorithm that rated the quality of each age based on criteria established by Rodríguez-Rey *et al*.^1^. Since the paper was published, we received some useful feedback to improve the R code to ensure an objective and consistent rating of ages, which are outlined below.

**Possible contamination of samples reported by authors**

In the previously published version of the algorithm detailed in Peters *et al*.^2^, we used details on potential sample contamination provided in the literature to inform the quality ranking. However, since these reports were too inconsistent across publications to provide objective quality control, we modified the algorithm not to use reported contamination for determining sample ranking. We now retain the information of reported contamination in the database as additional information for the end user to make final decisions on whether to accept or reject samples.

**Fish otoliths as material used for radiocarbon dating**

Fish otoliths are often used for radiocarbon dating; however, otoliths are not specifically mentioned in Table 2 of Rodríguez-Rey *et al*.^1^. Given that otoliths are aragonite carbonate samples, they should be grouped with shell and coral. We have now updated the algorithm to include otoliths in this group, which means the quality rating criteria outlined for this group also apply to otoliths.

**Missing data**

The authors of Gillespie *et al*.^3^ provided advice for dealing with missing and/or incorrect details of their published ages included in the *FosSahul* 2.0 database, mainly regarding dating material and pre-treatment. These have now been corrected. Furthermore, we have added six new ages from Gillespie *et al*.^4^ to the updated database.

**Figure correction**

Figure 3 in the original publication^2^ contained an error in the calculation of temperature and precipitation anomalies. We have corrected this error here (see Figure 1). Both the original and updated datasets and code are now hosted on figshare^5^ and GitHub (https://github.com/GlobalEcologyFlinders/FosSahul).

**Fig. 1:**
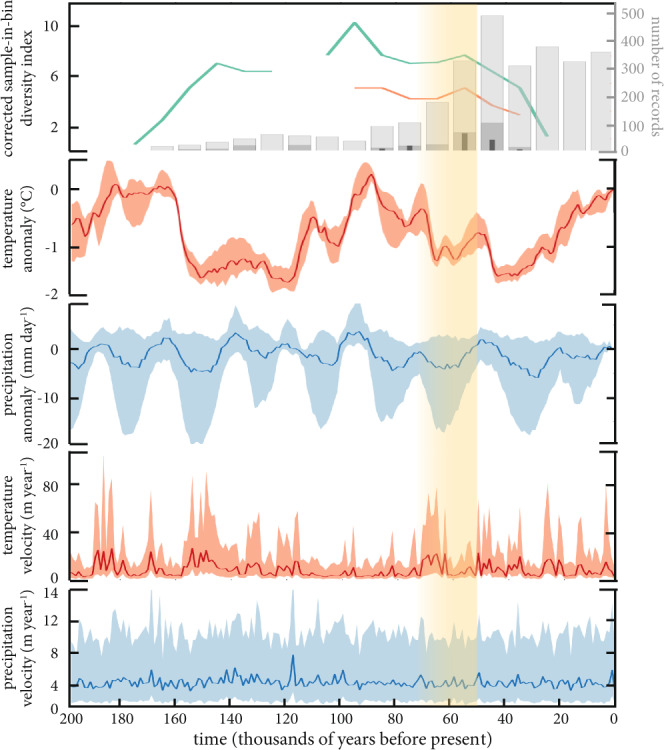
Genus-level corrected, sampled-in-bin diversity index calculated from *FosSahul* 2.0 high-quality ages (i.e., scored A* and A) for megafauna specimens (from Sahul = green; from south-eastern Australia = orange), number of megafauna records (based on the full dataset = light grey; high-quality ages only = dark grey; high- quality ages for south-eastern Australia only = thin black bars), mean annual temperature (°C) and precipitation anomaly (mm day^-1^) relative to the present day, temperature velocity (m year^-1^), and precipitation velocity (m year^-1^) across time (in thousands of years before present). Both the ‘corrected, sampled-in-bin diversity index’ and the ‘number of records’ are calculated using 10,000-year time increments, with the oldest records dated to 180,000 years before present. Climate variable plots show the median value (solid line), and the 25^th^ and 75^th^ percentiles (light shading) calculated across Sahul. Yellow shading represents putative arrival window (including uncertainties) of humans in Sahul; see Bradshaw *et al*.^6^ for discussion. This is a corrected version of Figure 3 from the original publication^2^ which contained an error in the calculation of temperature and precipitation anomalies.
